# Influence of Obstructive Apnea Index on Persistent Left Ventricular Dysfunction in Patients with ST-Segment Elevation Myocardial Infarction

**DOI:** 10.3390/jcm13040986

**Published:** 2024-02-08

**Authors:** Jin Kirigaya, Noriaki Iwahashi, Tomoaki Ishigami, Takeru Abe, Masaomi Gohbara, Yohei Hanajima, Mutsuo Horii, Kozo Okada, Yasushi Matsuzawa, Masami Kosuge, Toshiaki Ebina, Kiyoshi Hibi

**Affiliations:** 1 Department of Cardiology, Yokohama City University Medical Center, Yokohama 232-0024, Japan; kirigaya@yokohama-cu.ac.jp (J.K.); gocchi3@yokohama-cu.ac.jp (M.G.); hana0520@yokohama-cu.ac.jp (Y.H.); m9100239@yokohama-cu.ac.jp (M.H.); kokada2@yokohama-cu.ac.jp (K.O.); matsu@yokohama-cu.ac.jp (Y.M.); masami-kosuge@pop06.odn.ne.jp (M.K.); tebina@yokohama-cu.ac.jp (T.E.); 2 Department of Cardiology, Yokohama City University Hospital, Yokohama 236-0004, Japan; hibikiyo@yokohama-cu.ac.jp; 3 Advanced Critical Care and Emergency Center, Yokohama City University Medical Center, Yokohama 232-0024, Japan; abet@yokohama-cu.ac.jp

**Keywords:** left ventricular dysfunction, sleep-disordered breathing, ST-segment elevation myocardial infarction

## Abstract

**Background**: We retrospectively investigated the effects of the severity and classification of sleep-disordered breathing (SDB) on left ventricular (LV) function in patients with ST-segment elevation myocardial infarction (STEMI). **Methods**: A total of 115 patients with STEMIs underwent a sleep study using a multichannel frontopolar electroencephalography recording device (Sleep Profiler) one week after STEMI onset. We evaluated LV global longitudinal strain (LV-GLS) using two-dimensional echocardiography at one week and seven months. Patients were classified as no SDB (AHI < 5 events/h), obstructive SDB (over 50% of apnea events are obstructive), and central SDB (over 50% of apnea events are central). Due to the device’s limitations in distinguishing obstructive from central hypopnea, SDB classification was based on apnea index percentages. **Results**: The obstructive apnea index (OAI) was significantly associated with LV-GLS at one week (r = 0.24, *p* = 0.027) and seven months (r = 0.21, *p* = 0.020). No such correlations were found for the central apnea index and SDB classification. Multivariable regression analysis showed that the OAI was independently associated with LV-GLS at one week (β = 0.24, *p* = 0.002) and seven months (β = 0.20, *p* = 0.008). **Conclusions**: OAI is associated with persistent LV dysfunction assessed by LV-GLS in STEMI.

## 1. Introduction

Among patients with ST-segment elevation myocardial infarction (STEMI), those with sleep-disordered breathing (SDB) have a higher incidence of adverse cardiovascular events than those without SDB [1,2]. There are two main types of SDB: obstructive sleep apnea (OSA) and central sleep apnea (CSA). OSA was reported to be associated with left ventricular (LV) remodeling and infarct size after STEMI [3,4]. CSA was also reported as the prognostic factor of acute myocardial infarctions (AMI) [5,6,7]. However, the effect of CSA on LV function and structure following STEMIs remains unclear [4].

Recent studies have revealed that left ventricular global longitudinal strain (LV-GLS) measured by two-dimensional echocardiography (2DE) after STEMI is the most promising marker of LV dysfunction and a superior predictor of outcome compared to the conventional echocardiographic parameters of LV function, such as LV ejection fraction (LVEF) or the wall motion score index [8,9]. LV-GLS is useful in patients with STEMIs with preserved LVEF [10] but also in ischemic cardiomyopathy with reduced LVEF [11]; LV-GLS values represent total LV myocardial function, which includes infarct damage [8].

However, the effect of SDB on LV function assessed by LV-GLS after STEMI has yet to be investigated. This study aimed to identify the association between the severity and the type of SDB with serial LV-GLS after the first STEMI. Our findings would be beneficial for classifying more severely ill STEMI patients with SDB at an early stage.

## 2. Materials and Methods

### 2.1. Study Design

This study was a retrospective observational study. Between December 2016 and January 2020, 378 consecutive patients hospitalized for STEMIs at our medical center were recruited. We analyzed patients with a first incident of STEMI who underwent a sleep study and 2DE one week and 7 (±2) months after the onset of STEMI to examine the influence of SDB on LV dysfunction after STEMIs.

**Figure 1:**
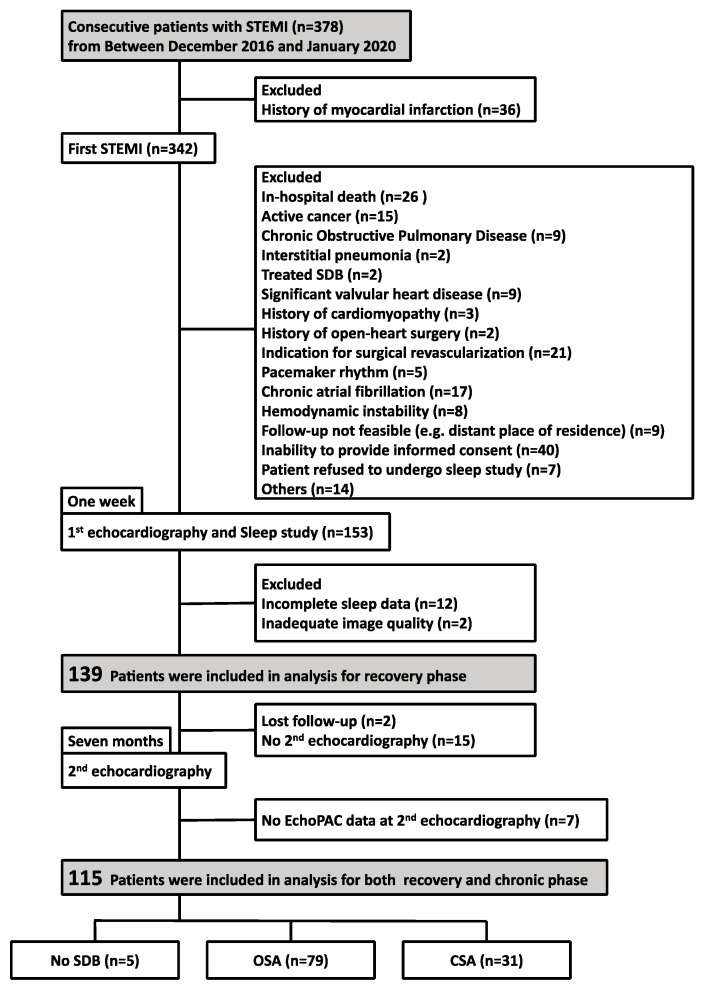
Study flow chart. Footnotes: CSA, central sleep apnea; OSA, obstructive sleep apnea; SDB, sleep-disordered breathing; STEMI, ST-segment elevation myocardial infarction.

Patients meeting any of the following criteria were excluded: in-hospital death, active cancer, treated SDB, chronic obstructive pulmonary disease, interstitial pneumonia, significant valvular heart disease, history of cardiomyopathy, history of open-heart surgery, indication for surgical revascularization, pacemaker rhythm, chronic atrial fibrillation, hemodynamic instability, unfeasible follow-up (e.g., residing in a distant location), inability to provide informed consent, refusal to undergo a sleep study, incomplete sleep data, inadequate image quality, lost to follow-up, no second echocardiography, and no EchoPAC data at second echocardiography. The eligibility criteria were met by 115 patients who were enrolled in the study (Figure 1). The study protocol was approved by the Ethics Committee of Yokohama City University (F221200035, 1 December 2023), and the study complied with the provisions of the Declaration of Helsinki. The ethics committee waived the requirement for individual informed consent because of the retrospective nature of the study and the availability of the patient data.

### 2.2. Clinical, Laboratory, and Instrumental Data

**Figure 2:**
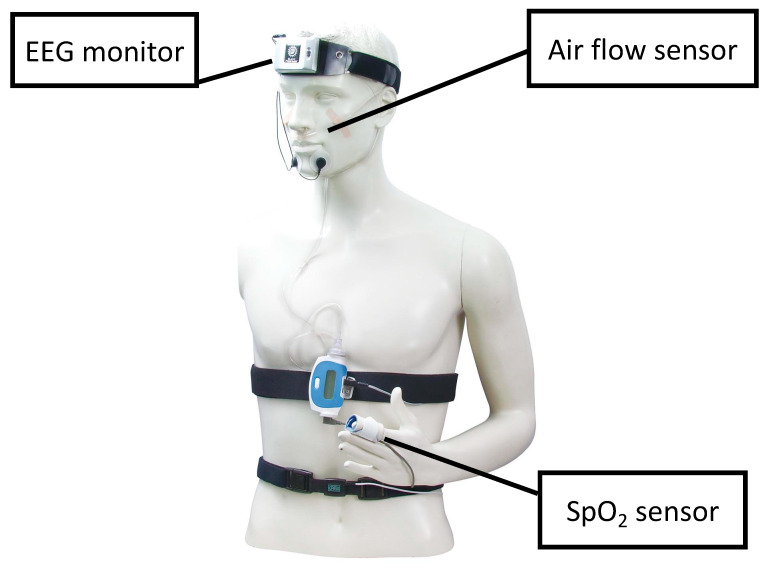
Sleep apnea testing using the Sleep Profiler. All subjects underwent polysomnography for one week with a Sleep Profiler, which is a wireless, self-application, ambulatory EEG sleep monitoring. Footnotes: EEG, electroencephalogram.

STEMI was defined as chest pain lasting at least 30 min with new ST-segment elevation and an elevated cardiac troponin I level > 99th percentile of a standard population reference [12]. The following criteria were used to define ST-segment elevation: new ST-segment elevation at J in at least two contiguous leads of 0.2 mV in men or 0.15 mV in women in leads V2–V3, or 0.1 mV in other leads. New left bundle branch block was also considered as STEMI. Treatment of all patients followed the current guidelines of the Japanese Circulation Society [13]. For one week, a sleep study using multichannel frontopolar electroencephalography recordings was performed using a forehead-worn recording device (Sleep Profiler, Advanced Brain Monitoring, Carlsbad, CA, USA) (Figure 2) [14,15]. Sleep profilers are ambulatory sleep electroencephalography (EEG) devices that can assess sleep architecture and continuity with acceptable comparability to the gold standard laboratory procedure of polysomnography [14]. The Sleep Profiler was applied to the forehead at approximately 9:00 p.m., and the device was removed in the morning at a convenient time. Recordings were transferred to the portal, and software was used to verify signal quality and automatic staging visually. The portable monitor recorded the nasal pressure, chest movements, snoring, body position, activity, pulse oximetry, heart rate, and oxygen saturation signals. The data from the Sleep Profiler were manually scored using 11 sleep technicians blinded to the clinical data. All technicians had more than three years of experience performing these analyses. Definitions and scoring methods were based on the American Academy of Sleep Medicine (AASM) version 2.1 [16] Apnea and hypopnea events were quantified, and SDB severity was assessed using the frequency of apnea and hypopnea events per hour of sleep (AI [apnea index]; HI [hypopnea index]) [16]. Obstructive and central AI scores were computed separately. We defined the obstructive apnea index (OAI) and central apnea index (CAI) as the mean number of obstructive apneas and central apnea. Since the Sleep Profiler^®︎^ cannot discriminate between obstructive and central sleep disturbance for hypopnea, the classification was based on the percentage of apnea index. The patients were divided into three groups based on the following specifications: no SDB (AHI < 5 events/h), an OSA-dominant group (≥50% of apnea events obstructive), or a CSA-dominant group (>50% of apnea events central) [16].

A standardized 2DE study was performed by experienced sonographers using commercially available ultrasound systems (Vivid q, Vivid E9, or VividE95; GE Healthcare, Chicago, IL, USA) with 3.5 MHz or M5S transducers at the same time as the sleep study, one week and 7 (±2) months after the onset of STEMI. It is recognized that LV remodeling after AMI is generally considered to be substantially complete in approximately 90% of cases at approximately six months after onset [17,18]. Thus, we adopted 2DE at seven months to evaluate LV functional recovery. Standard 2D, color, continuous-wave, pulsed-wave, and Doppler images were acquired and stored in cine-loop format. Two-dimensional parasternal long-axis images determined LV cavity dimensions and wall thickness. The following LV diastolic parameters were measured: peak early (E) and late (A) diastolic velocities and E-wave deceleration times by pulsed-wave Doppler of transmitral flow. Tissue Doppler images of the left ventricle were obtained in the apical four-chamber view at the end of expiration, and the peak early diastolic myocardial velocity (E’) was measured at the base of the septum and the base of the lateral mitral annulus. The ratio of peak transmitral E-wave/septal e’ (E/e’ sep), the ratio of peak transmitral E-wave/lateral e’ (E/e’ lat), and the mean of E/e’ sep and E/e’ lat (E/e’ mean) were assessed. LVEF and left atrial volume were measured using the disk-summation method in the apical four-chamber and two-chamber views. LV end-diastolic and end-systolic volume and left atrial volume were indexed to body surface area.

Two-dimensional echocardiographic data were also analyzed offline (EchoPAC PC; GE Healthcare) by an experienced investigator blinded to baseline clinical data. LV-GLS analysis was performed by speckle tracking in three apical windows: apical longitudinal long-axis views, four-chamber views, and two-chamber views. A semi-automated function tracked the ventricular endocardium, and manual point-and-click adjustments were made if the tracking was inaccurate. Every projection covered six segments, resulting in 18 segments for the LV-GLS calculation. LV-GLS was calculated by the software as the average of the systolic longitudinal strain peaks of the three apical views. If systolic longitudinal strain peaks could only be assessed in two of the three apical views, the average of the longitudinal strain peaks in the two views was used to calculate the LV-GLS. Exclusions of segments due to obscuration by rib artifacts or lung tissue were made at the discretion of the analyses.

### 2.3. Statistical Analyses

Categorical variables are expressed as frequencies and percentages, and continuous data are expressed as the mean ± standard deviation or as medians and interquartile ranges. The groups of patients with no SDB, OSA, or CSA were compared using the Kruskal–Wallis test for continuous variables and a chi-square test or Fisher’s exact test for categorical variables. Univariate regression analyses were performed to predict the LV-GLS at 1 week and 7 months. Peak creatine kinase muscle and brain isoenzyme (CK-MB) (IU/l) levels, reperfusion within 12 h from onset of STEMI, culprit left descending artery, multi-vessel disease, age, final thrombolysis in myocardial infarction (TIMI) flow grade = 3, initial TIMI flow grade ≥ 2, AHI, OSA predominance, CSA-predominant, OAI, CAI, and CSR, all of which were considered important predictors of LV function, were included [19,20]. Next, univariate predictors of LV-GLS with *p* < 0.05 were entered into a multiple regression analysis using a forward stepwise algorithm (Models I, II, and III). AHI and apnea indexes were analyzed separately to avoid multicollinearity. All statistical analyses were performed using JMP pro 15.0 software (SAS Institute, Cary, NC, USA). For all analyses, *p* < 0.05 was considered to indicate statistical significance. The datasets produced or analyzed during this study are not publicly available due to privacy and ethical restrictions. These data are available from the corresponding author on reasonable request.

## 3. Results

### 3.1. Baseline Characteristics of Subjects

Table 1 shows the characteristics of the patients. The patients had an average age of 65 years (±11), and the majority were male (87%). The median peak CK-MB level was 198 IU/L (124–363). Differences in patient characteristics among the three groups were not observed, except that the frequency of females was relatively high in the CSA group. Sleep parameters in the three groups are shown in Table 1. Sleep stage did not vary amongst the four groups but showed less N3 stage overall. CSR was significantly more frequent in the CSA group.

### 3.2. Relationship between SDB and 2DE Parameters

Table 2 presents the baseline and follow-up 2DE parameters. The three groups demonstrated no significant differences in baseline LV end-diastolic volume index (LVEDVI), LVEF, LV-GLS, or other parameters. Next, we examined the relationship between the two apnea indices and the echocardiographic parameters (Table 3). The OAI was significantly associated with E/A, E/e’, and LV-GLS at one week. However, the CAI was not associated with these parameters. At seven months, the OAI was significantly associated with LVEDVI, LVESVI, E/A, and LV-GLS, whereas the CAI was not.

We have shown the relationship between LV functional improvement and sleep parameters. LV functional improvement was defined as the absolute difference between chronic and recovery LV-GLS greater than the median (0.9). We found no significant association between LV functional improvement and sleep parameters (Table 4).

### 3.3. Multiple Regression Analyses for the Prediction of LV-GLS at One Week and Seven Months

The apnea index was hypothesized to be associated with LV-GLS after STEMI, and univariate and multiple regression analyses were performed to predict LV-GLS at one week and seven months. Regression analysis identified OAI and AHI as independent predictors of LV-GLS at one week (Models II and III in Table 5) (β coefficient = 0.24, *p* = 0.002; and β coefficient = 0.26, *p* < 0.001, respectively). OAI and AHI were also independent predictors of LV-GLS at seven months in a similar analysis (Models II and III in Table 6) (β coefficient = 0.20, *p* = 0.008; and β coefficient = 0.22, *p* = 0.004, respectively).

## 4. Discussion

This study investigated the effect of SDB on LV function, assessed using LV-GLS after the first STEMI. Our results showed that OAI and AHI were independent predictors of persistent LV dysfunction assessed using LV-GLS after the first STEMI, whereas the classifications of SDB and CAI were not. This is the first study to demonstrate the effect of OAI on LV function measured by LV-GLS, a superior cardiac function index compared to conventional indices [8,9], after STEMI. In addition, the study presents the novel finding that obstructive apnea severity, rather than SDB classification, is associated with reduced LV systolic function after STEMI.

There have been reports of the relationship between OSA and cardiac functional recovery after STEMIs. OSA adversely affects cardiac function through increased cardiac afterload [21], stimulation of sympathetic nerve activity [21], and induction of myocardial ischemia due to hypoxia [22]. In the early setting after STEMI, the heart may be vulnerable and susceptible to the adverse effects of OSA, including endothelial dysfunction and increased cardiac workload [23]. Indeed, previous studies have shown that patients with STEMI and OSA experience prolonged myocardial ischemia [24], less salvaged myocardium [3], and adverse LV remodeling [24] compared to those without OSA. These factors increase the risk of persistent LV dysfunction.

Our study showed that OAI was significantly associated with E/e’ at one week, which is the parameter of LV filling pressure. The association between OAI and E/e’ at seven months also approached significance (*p* = 0.057). A previous study demonstrated that obstructive apnea induces elevation of the LV filling pressure by increasing the LV afterload [25,26]. Increased LV wall stress induced by high LV filling pressure enhances myocardial cell death due to the architectural rearrangement of myocytes [27]. These results suggest that a continuous increase in LV wall stress caused by a high OAI may have induced persistent LV systolic dysfunction after STEMI.

In our study, OAI significantly correlated with a persistent decrease in LV-GLS. In contrast, CAI did not influence the LV-GLS. This mechanism can be explained as follows: the key pathophysiological differences between patients with CSA and OSA are reported to be negative intrathoracic pressure swings in the OSA group owing to respiratory effort against the occluded pharynx, raised blood pressure in the OSA cohort, and arousals [23]. In reality, increased LV transmural pressure in patients with OSA was reported to promote spherical cardiac remodeling and thinning of the LV wall in the region of the myocardial infarction [4]. Therefore, it is reasonable that OAI, but not CAI, is associated with persistent deterioration of LV function after STEMI.

Previous studies have shown mixed results on the relationship between the severity and classification of SDB and LV function after STEMIs, partly because of differences in the definition of SDB and the techniques for assessing cardiac function. Buchner et al. reported that patients with SDB had significantly lower LVEFs 3 months after the onset of STEMIs, as assessed by cardiac magnetic resonance imaging, than patients without SDB (LVEF = 48% vs. LVEF = 54%, *p* = 0.023) [3]. They reported that both OSA and CSA were associated with less myocardial salvage and a minor reduction in infarct size. However, in their study, the effects of OSA and CSA on LV functional recovery were not separately investigated. Additionally, LVEF, which is inferior to LV-GLS as a prognostic marker of STEMI, was used as a parameter for LV systolic function. Fisser et al. reported that increased LV transmural pressures in patients with OSA, but not in patients with CSA, may cause spherical cardiac remodeling and thinning of the LV wall [4]. Their study assessed spherical remodeling, calculated according to the sphericity index, by cardiac magnetic resonance imaging at baseline and three months after AMI. In contrast to CSA, OSA severity was independently associated with increased systolic sphericity index. Their results are consistent with ours in that only OSA, and not CSA, affects cardiac function. Our findings enhance the results of Fisser et al.’s study and provide a deeper understanding of the relationship between the OSA severity and LV functional recovery after STEMIs because we examined LV function using LV-GLS, the most promising marker of LV dysfunction and a superior predictor of outcome compared to the conventional LV functional parameters [8,9].

Previous studies have classified SDB according to the ratio of the central hypo-apnea index and obstructive hypo-apnea index to total AHI, and the influence of SDB on cardiac function was discussed by SDB classification (OSA-dominant or CSA-dominant) [3]. However, CSA and OSA can coexist [28,29]. In other words, even in patients classified as having CSA, the OAI may be high in some cases if the AHI is high. Therefore, to evaluate the relationship between SDB and STEMI, it may be beneficial to assess each apnea index rather than simply classify SDB into two types.

The results from the current study indicate that OAI influences LV function in the chronic phase after STEMI. A decreased LV systolic function during this phase could be a poor prognostic factor [30]. Hence, more aggressive treatment with high-dose renin-angiotensin system inhibitors, beta-blockers, or sodium-glucose cotransporter-2 inhibitors and closer follow-up of patients with high OAI early after the onset of STEMI may be required. In our study, AHI was also an independent factor associated with recovery and chronic-phase LV systolic dysfunction. However, measurement of AHI alone may not identify OAI, a potential therapeutic factor associated with cardiac dysfunction in AMI. Therefore, not only AHI but also CAI and OAI should be assessed separately in patients with STEMI.

Our results may have important implications when considering the indication for continuous positive airway pressure (CPAP) therapy in STEMI complicated by SDB. Determining the indications for CPAP during STEMI recovery is often difficult. Recent randomized controlled trials have failed to demonstrate the beneficial prognostic impact of CPAP treatment in the primary or secondary setting of AMI [31,32]. In the present study, we demonstrated that the OAI predicts impaired recovery of LV-GLS. This indicates that relief from OSA may have facilitated the recovery of LV function. CPAP treatment in patients with high OAI may improve LV function after STEMI.

### Study Limitations

This study had several limitations. Firstly, this was a single-center retrospective study, and only patients experiencing a first STEMI who consented to a sleep study and for whom echocardiography could be performed in both the recovery and chronic phases were included. Therefore, the current study is limited by a small sample size. Thus, the potential relationship between CSA and cardiac function may not have been fully explored. However, our detailed analysis of the relationship between SDB and cardiac function using LV-GLS is novel and sets this study apart from its predecessors. We believe that further research involving a larger patient cohort is needed to investigate these associations. Secondly, our study showed that OAI was negatively associated with LV-GLS in both the recovery and chronic phases. However, OAI was not associated with LV-GLS recovery (Table 4). The study included patients eligible for a sleep study with no cardiovascular events up to the time of the chronic phase echocardiogram. Consequently, the patient cohort was small, and the infarct size was relatively modest. These factors may have prevented us from elucidating the relationship between sleep parameters and LV-GLS recovery in STEMI. Future studies involving larger patient populations may be able to investigate the relationship between sleep parameters and LV-GLS recovery in STEMI. Thirdly, all sleep studies were performed using Sleep Profiler rather than conventional polysomnography, the accepted gold standard for determining the presence and severity of SDB. Fourthly, since the Sleep Profiler cannot discriminate between obstructive hypopnea and central hypopnea, the classification was based on the percentage of the apnea index. The classification of hypopneas as central or obstructive is often difficult and has not been clearly defined in clinical studies [33]. Therefore, we believe that the fact that we were only able to examine obstructive and central apneas in this study does not diminish the significance of the study. However, for further understanding of the pathophysiology, it would be desirable to include hypopneas in future studies. Finally, because of this device limitation, we could not evaluate whether obstructive or hypopnea affected cardiac functions as obstructive apnea does.

## 5. Conclusions

OAI is associated with persistent LV dysfunction in patients with STEMI. Our findings would be beneficial for classifying more severely ill patients with STEMI at an early stage. Careful echocardiographic assessment and more aggressive treatment with medication should be provided to patients with STEMI and a higher OAI.

## Figures and Tables

**Table 1:** Baseline characteristics.

Parameter	No SDB (n = 5)	OSA (n = 79)	CSA (n = 31)	*p* Value
Age, years	70 ± 12	66 ± 10	62 ± 13	0.260
Male, n (%)	3 (60)	68 (86)	30 (96)	0.045
Body mass index, kg/m^2^	21.9 ± 1.6	24.8 ± 4.5	25.4 ± 3.9	0.168
Killip class ≥ II, n (%)	1 (20)	13 (16)	3 (9)	0.629
Hypertension, n (%)	2 (40)	52 (65)	19 (61)	0.486
Diabetes mellitus, n (%)	2 (40)	28 (35)	10 (32)	0.922
Dyslipidemia, n (%)	4 (80)	61 (77)	23 (74)	0.928
Current smoker, n (%)	1 (20)	23 (29)	16 (51)	0.064
Reperfusion within 12 h from onset of STEMI, n (%)	5 (100)	70 (88)	30 (96)	0.306
Culprit LAD, n (%)	3 (60)	50 (63)	12 (38)	0.064
Multi-vessel disease, n (%)	4 (80)	28 (35)	9 (29)	0.087
Peak CK-MB, IU/L	218 (170–548)	198 (124–363)	182 (90–361)	0.6
eGFR, mL/min/1.73 m^2^	48 (44–67)	64 (53–78)	61 (54–75)	0.272
Medication at discharge, n (%)				
β-blocker	2 (40)	50 (64)	22 (70)	0.404
ACE-I/ARB	4 (80)	68 (86)	27 (87)	0.913
Statin	5 (100)	78 (98)	28 (90)	0.087
Aspirin	4 (80)	74 (93)	30 (96)	0.342
Arousal index	8.8 (7.1–10.5)	19.9 (12.9–28.2)	18.7 (12.1–22.2)	0.013
Mean SpO_2_, %	98 (97–98)	97 (95–98)	97 (95–98)	0.115
Minimum SpO_2_, %	93 (63–93)	83 (67–86)	77 (69–87)	0.184
3% ODI	4.3 (1.8–16.1)	31.7 (17.8–51.1)	33.6 (14.4–57.4)	0.017
Sleep stage (% sleep time)				
N1	13.9 (7.1–31.3)	30.5 (21.6–43.9)	19.6 (43.7–27.1)	0.173
N2	51.4 (38.9–63.4)	46.8 (34.4–57.3)	47.0 (25.8–52.4)	0.713
N3	3.8 (0.1–13.8)	0 (0–0.9)	0 (0–0.5)	0.229
REM	23.9 (21.7–26.9)	18.6 (13.8–26.3)	19.2 (16.9–24.2)	0.292
Obstructive apnea index	0 (0–0.4)	8.6 (3.4–20)	2 (0.6–5.7)	<0.001
Central apnea index	0 (0–0.8)	0.3 (0–1.9)	4.3 (1.5–19.8)	<0.001
Total apnea index	0.4 (0.2–0.9)	11.7 (3.9–22.6)	9.4 (2.1–31.1)	0.048
Hypopnea index	3 (1.6–3.9)	12.7 (9.0–19.1)	10.6 (6.8–18.2)	0.026
Apnea hypopnea index	3.9 (1.9–4.2)	29.3 (17.6–46.4)	27.5 (15.9–46.8)	0.001
Cheyne stokes respiration, %	0 (0)	7 (9)	15 (48)	<0.001

Data were expressed as a mean (standard deviation) or a median (interquartile range) for continuous variables, or as a frequency (percentage) for categorical variables. ACE-I, angiotensin-converting-enzyme inhibitor; ARB, angiotensin-II receptor blocker, CK-MB, creatine kinase muscle and brain isoenzyme; CSA, central sleep apnea; eGFR, estimated glomerular filtration rate; LAD, left anterior descending coronary artery; ODI, oxygen desaturation index; OSA, obstructive sleep apnea; REM, rapid eye movements; SDB, sleep discorded breathing.

**Table 2:** Two-dimensional echocardiography at one week and seven months.

Parameter	No SDB (n = 5)	OSA (n = 79)	CSA (n = 32)	*p*-Value
Echocardiography at 1 week				
LVEF; %	50 (31–54)	49 (44–55)	50 (43–54)	0.604
LVEDVI (mL/m^2^)	58 (49–68)	59 (45–68)	54 (43–71)	0.961
LVESVI (mL/m^2^)	24 (19–45)	28 (21–37)	26 (21–36)	0.732
Deceleration time; msec	193 (156–234)	181 (152–216)	181 (144–224)	0.937
E/A	0.85 (0.59–1.21)	0.82 (0.66–1.12)	0.76 (0.65–0.99)	0.877
E/e’ (mean)	11.3 (8.3–14.6)	10.2 (8.6–12.9)	9.6 (7.2–11.7)	0.342
LAVI (mL/m^2^)	35.7 (29.4–51.2)	33.2 (26.8–41.5)	30.8 (26.9–37.7)	0.500
LV-GLS	−14.7 (−15.8–−10.9)	−13.3 (−15.3–−11.2)	−13.4 (−15.0–−12.6)	0.816
Echocardiography at 7 months				
LVEF; %	47 (44–57)	51 (44–57)	51 (46–58)	0.435
LVEDVI (mL/m^2^)	68 (48–70)	62 (50–79)	58 (46–74)	0.563
LVESVI (mL/m^2^)	37 (24–42)	31 (24–39)	25 (23–38)	0.387
Deceleration time; msec	224 (180–314)	214 (179–260)	251 (201–285)	0.124
E/A	0.82 (0.52–1.25)	0.72 (0.61–0.89)	0.80 (0.71–0.90)	0.320
E/e’ (mean)	10.3 (6.7–12.0)	9.0 (6.8–11.5)	9.9 (7.1–11.3)	0.979
LAVI (mL/m^2^)	40.0 (31.4–48.3)	31.1 (25.5–40.0)	31.0 (27.8–42.3)	0.190
LV-GLS	−13.8 (−19.0–−11.0)	−14.9 (−16.2–−11.7)	−15.0 (−16.1–−13.1)	0.938

Data were expressed as a median (interquartile range) for continuous variables. LAVI, left atrial volume index; LVEF, left ventricular ejection fraction; LVEDVI, left ventricular end-diastolic volume index; LVESVI, left ventricular end-systolic volume index; LV-GLS, left ventricular global longitudinal strain.

**Table 3:** The relationship between apnea index and echocardiographic parameters.

Parameter	Obstructive Apnea Index		Central Apnea Index	
	R	*p*-Value	R	*p*-Value
Echocardiography at 1 week				
LVEF; %	−0.15	0.105	−0.02	0.799
LVEDVI (mL/m^2^)	0.11	0.214	0.09	0.308
LVESVI (mL/m^2^)	0.16	0.069	0.05	0.550
Deceleration time; msec	0.01	0.836	0.09	0.326
E/A	−0.21	0.025	−0.11	0.241
E/e’ (mean)	0.21	0.024	0.004	0.959
LAVI (mL/m^2^)	−0.03	0.730	−0.02	0.792
LV-GLS	0.24	0.007	0.01	0.893
Echocardiography at 7 months				
LVEF; %	−0.17	0.064	−0.05	0.534
LVEDVI (mL/m^2^)	0.22	0.014	0.11	0.209
LVESVI (mL/m^2^)	0.26	0.004	0.10	0.277
Deceleration time; msec	0.007	0.936	0.08	0.362
E/A	−0.30	0.001	−0.02	0.787
E/e’ (mean)	0.17	0.057	0.13	0.146
LAVI (mL/m^2^)	0.006	0.943	0.15	0.098
LV-GLS	0.21	0.020	0.04	0.619

LAVI, left atrial volume index; LVEF, left ventricular ejection fraction; LVEDVI, left ventricular end-diastolic volume index; LVESVI, left ventricular end-systolic volume index; LV-GLS, left ventricular global longitudinal strain.

**Table 4:** The association between LV-GLS improvement and sleep parameters.

Variables	Univariable	
	Odds Ratio	*p*-Value
Arousal index	1.00 (0.97–1.03)	0.837
Mean SpO_2_; %	0.80 (0.63–1.02)	0.081
Minimum SpO_2_; %	0.99 (0.97–1.01)	0.653
3% ODI	1.00 (0.99–1.02)	0.635
Sleep stage N1 (%sleep time)	1.01 (0.99–1.03)	0.063
N2	0.98 (0.96–1.00)	0.011
N3	0.89 (0.77–1.02)	0.328
REM	1.00 (0.96–1.04)	0.842
OSA predominant	0.74 (0.33–1.63)	0.458
CSA predominant	1.52 (0.66–3.49)	0.319
Obstructive apnea index	1.00 (0.98–1.03)	0.527
Central apnea index	1.00 (0.96–1.04)	0.875
Total apnea index	1.00 (0.98–1.02)	0.555
Hypopnea index	0.99 (0.96–1.03)	0.933
Apnea hypopnea index	1.00 (0.98–1.02)	0.634
Cheyne stokes respiration; %	1.22 (0.48–3.11)	0.668

CSA, central sleep apnea; ODI, oxygen desaturation index; OSA, obstructive sleep apnea; LV-GLS, left ventricular global longitudinal strain; REM, rapid eye movements.

**Table 5:** Univariate and multiple regression analyses for the prediction of LV-GLS at 1 week.

Variables	Univariate		Multivariate Model I		Multivariate Model II		Multivariate Model III	
	β	*p*-Value	β	*p*-Value	β	*p*-Value	β	*p*-Value
Peak CK-MB; per 1 IU/L	0.46	<0.001	0.30	0.001	0.26	0.004	0.27	0.002
Reperfusion within 12 h from onset of STEMI	0.13	0.137						
Culprit LAD	0.2	0.027	−0.15	0.063	−2.08	0.040	−0.16	0.038
Multi-vessel disease	−0.01	0.907						
Age; per year	0.01	0.899						
Final TIMI flow grade = 3	−0.13	0.159						
Initial TIMI flow grade ≥ 2	−0.41	<0.001	0.29	<0.001	0.37	<0.001	0.31	<0.001
AHI	0.25	0.006					0.26	<0.001
OSA predominant	−0.05	0.536						
CSA predominant	0.05	0.585						
Obstructive apnea index	0.21	0.020			0.24	0.002		
Central apnea index	0.01	0.893						
Cheyne stokes respiration	0.04	0.643						
Minimum SpO_2_	−0.18	0.053						
Mean SpO_2_	−0.03	0.710						
Oxygen desaturation index 3%	0.31	<0.001						
R^2^			0.295		0.354		0.298	
Adjusted R^2^			0.276		0.330		0.272	

AHI, apnea-hypopnea index; CK-MB, creatine kinase muscle and brain isoenzyme; CSA, central sleep apnea; LAD, left anterior descending coronary artery; OSA, obstructive sleep apnea; LV-GLS, left ventricular global longitudinal strain; TIMI, thrombolysis in myocardial infarction; STEMI, ST-segment elevation myocardial infarction.

**Table 6:** Univariate and multiple regression analyses for the prediction of LV-GLS at seven months.

Variables	Univariate		Multivariate I		Multivariate II		Multivariate III	
	Β	*p*-Value	β	*p*-Value	β	*p*-Value	β	*p*-Value
Peak CK-MB; per 1 IU/L	0.45	<0.001	0.26	0.004	0.23	0.011	0.24	0.007
Reperfusion within 12 h from onset of STEMI	0.17	0.059						
Culprit LAD	−0.23	0.011	−0.17	0.034	−0.18	0.021	−0.19	0.017
Multi-vessel disease	−0.02	0.773						
β-blocker on discharge	−0.23	0.013	0.08	0.297	0.07	0.352	0.06	0.439
Age; per year	0.04	0.604						
Final TIMI flow grade = 3	0.23	0.012	0.20	0.012	0.20	0.010	0.20	0.007
Initial TIMI flow grade ≥ 2	0.38	<0.001	0.25	0.006	0.20	0.001	0.28	0.001
AHI	0.21	0.021					0.22	0.004
OSA predominant	−0.05	0.530						
CSA predominant	0.04	0.638						
Obstructive apnea index	0.21	0.020			0.20	0.008		
Central apnea index	0.04	0.619						
Cheyne stokes respiration	−0.07	0.407						
Minimum SpO_2_	−0.15	0.103						
Mean SpO_2_	0.21	0.116						
Oxygen desaturation index 3%	0.24	0.008						
R^2^			0.327		0.369		0.376	
Adjusted R^2^			0.296		0.334		0.341	

AHI, apnea-hypopnea index; CK-MB, creatine kinase muscle and brain isoenzyme; CSA, central sleep apnea; LAD, left anterior descending coronary artery; OSA, obstructive sleep apnea; LV-GLS, left ventricular global longitudinal strain; STEMI, ST-segment elevation myocardial infarction; TIMI, thrombolysis in myocardial infarction.

## Data Availability

The datasets used and/or analyzed during the current study are available from the corresponding author on reasonable request.
